# Kissing intravascular lithotripsy in iliac in-stent restenosis related to underexpanded stents

**DOI:** 10.1016/j.jvscit.2024.101507

**Published:** 2024-04-16

**Authors:** Nicola Troisi, Francesco Canovaro, Daniele Adami, Vittorio Malquori, Raffaella Berchiolli

**Affiliations:** Vascular Surgery Unit, Department of Translational Research and New Technologies in Medicine and Surgery, University of Pisa, Pisa, Italy

**Keywords:** Chronic limb-threatening ischemia, In-stent restenosis, Intravascular lithotripsy

## Abstract

Intravascular lithotripsy (IVL) has been used for the treatment of native highly calcified arterial lesions. No data are available in the literature about its use in the treatment of noncoronary in-stent restenosis (ISR). We report the case of kissing IVL in highly calcified iliac ISR related to underexpansion of stents previously deployed in the common iliac arteries. The procedures were performed with a combined percutaneous right femoral and surgical left axillary access. This case demonstrates the safety and effectiveness of IVL even for the treatment of iliac ISR when other “standard” techniques cannot be used to obtain a satisfactory outcome. This technique needs to be evaluated further with multicenter experiences and adequate population sizes.

Complex high-grade calcified lesions are very common in patients with chronic limb-threatening ischemia.^1^ Arterial calcification still has a great role in the early and long-term outcomes of infrainguinal revascularizations.^2^ In addition, arterial calcification seems to be a risk factor for in-stent restenosis (ISR) in complex femoropopliteal lesions.^3^ Intravascular lithotripsy (IVL; Shockwave Medical Inc) seems to be a game changer in treating highly calcified coronary and noncoronary arterial lesions.^4^ Its role in the treatment of calcified ISR in the coronary arteries is currently debated in the literature.^5^ However, to the best of our knowledge, no study has reported on the treatment of calcified ISR in the lower limb peripheral arteries. In addition, underexpansion of the stents is a well-known phenomenon, especially in the coronary arteries.^6^^,^^7^

We report the case of kissing IVL in highly calcified iliac ISR related to underexpansion of stents previously deployed in the common iliac arteries. The patient provided written informed consent for the report of his case details and imaging studies.

## Case report

A 46-year-old male patient was admitted at our outpatient service with chronic limb-threatening ischemia (bilateral Rutherford class 5, bilateral WIfI [wound, ischemia, foot infection] classification 120). The preoperative toe brachial index was 0.4 in the right foot and 0.2 in the left foot. His medical history was notable for nicotine abuse, hyperlipidemia, hypertension, end-stage renal disease with lifelong dialysis, coronary artery disease with previous coronary artery bypass grafting, and implantation of an implantable cardioverter defibrillator. In addition, 3 years before admission, the patient underwent bilateral femoral endarterectomy with patch closure, kissing stenting with bare metal stents of the common iliac arteries, and an adjunctive stent in the right external iliac artery. Physical examination revealed absent femoral pulses (0 for both sides) and a good left radial pulse (4+).

**Fig 1:**
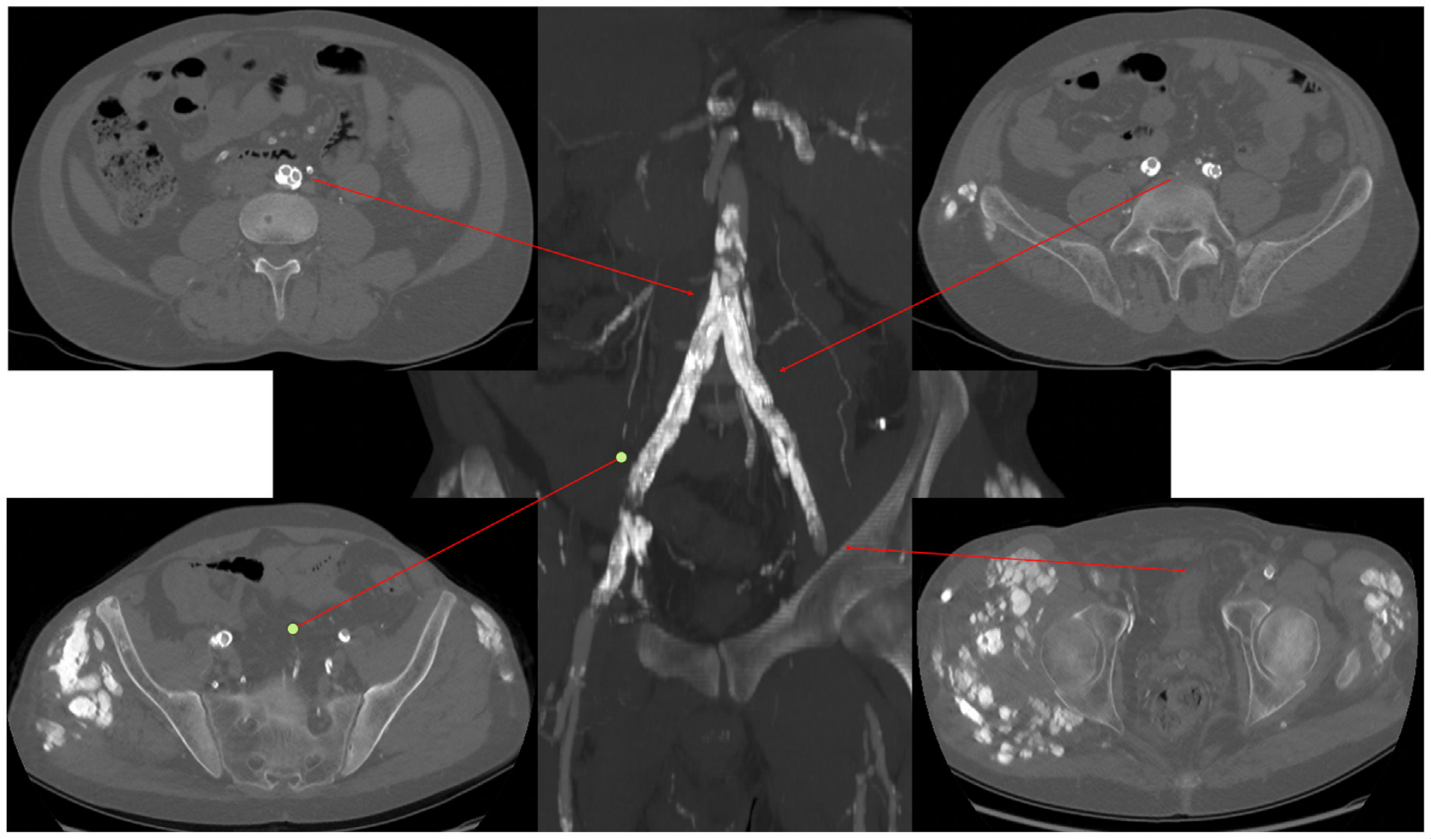
Preoperative computed tomography (CT) scan.

Preoperative duplex ultrasound detected bilateral iliac ISR. Computed tomography (CT) angiography confirmed the findings (Fig 1). The images revealed that iliac ISR (90% for both sides) was associated with insufficient expansion of the previously implanted stents due to high-grade calcified eccentric plaques. Regarding the outflow vessels, CT scans showed the absence of restenosis in both common femoral arteries and multiple stenoses of the infrainguinal vessels. A multistep treatment plan was made for the patient. As a first stage, we decided to treat the iliac ISR using IVL. After the initial procedure, the infrainguinal vessels will be reassessed to evaluate further treatment options.

**Fig 2:**
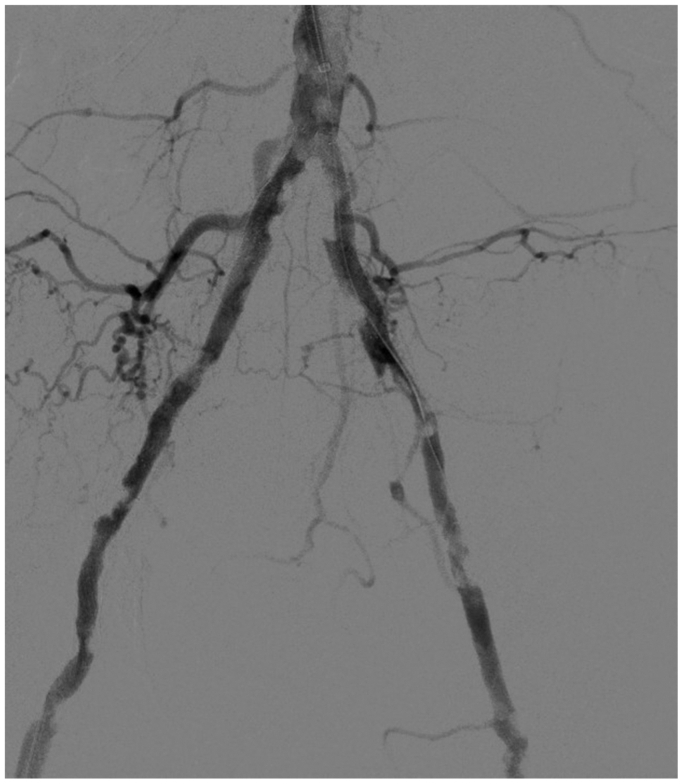
Preoperative angiography.

**Fig 3:**
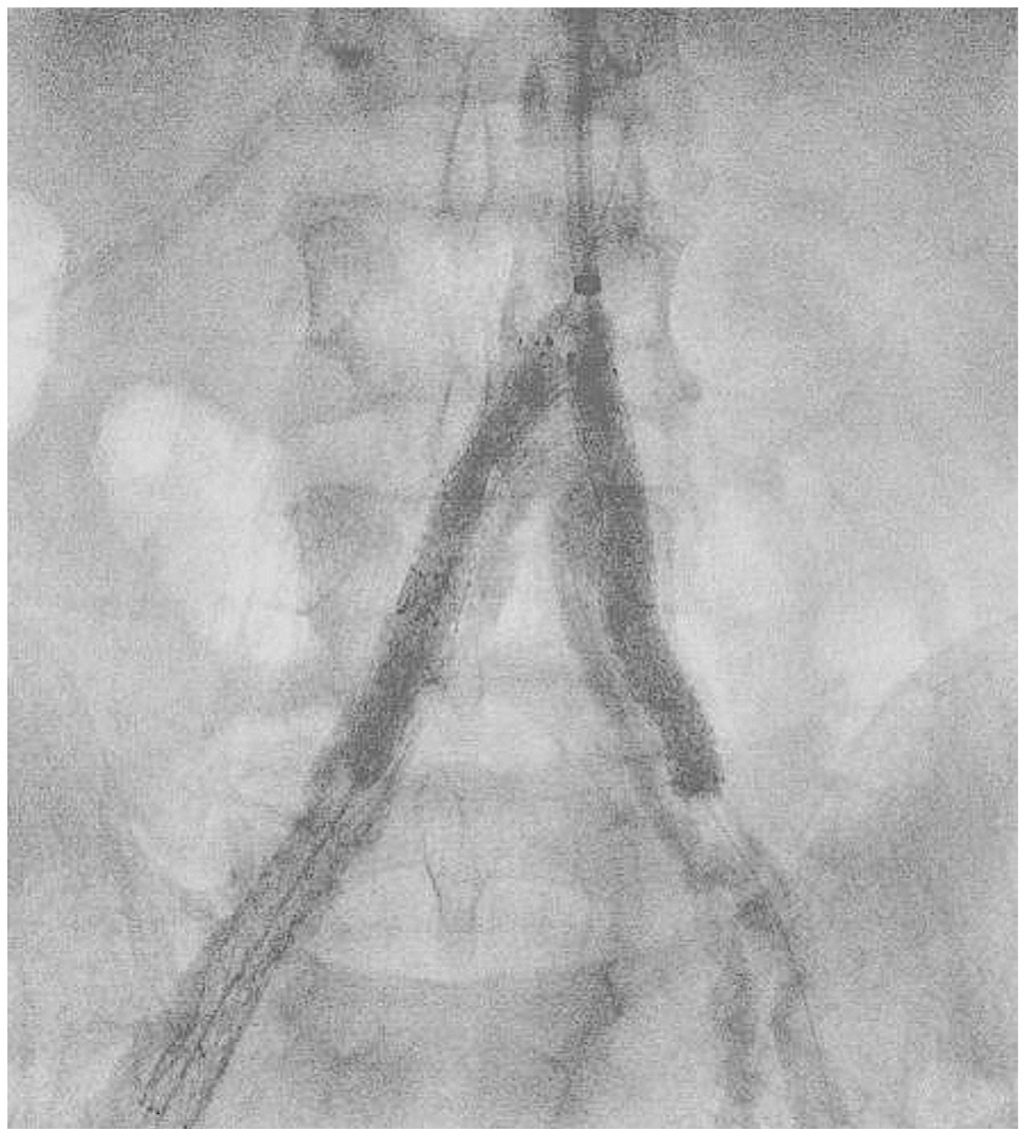
Kissing iliac intravascular lithotripsy (IVL).

**Fig 4:**
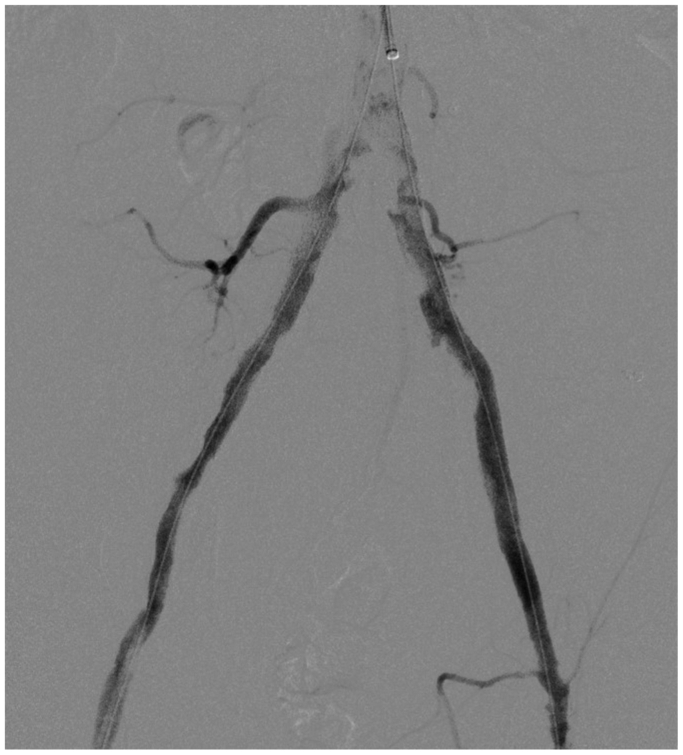
Postoperative angiography.

Retrograde echo-guided percutaneous access of the right common femoral artery was performed (8F sheath). Surgical dissection of the left axillary artery was performed. From above, an 8F, 70-cm sheath (Flexor; Cook Medical Inc) was placed into the abdominal aorta (Fig 2). Antegrade intraluminal crossing of the ISR in the left common iliac artery was obtained with a 0.035-in. guidewire (Advantage; Terumo Interventional Systems). The right iliac ISR was intraluminally crossed from below with a J-stiff, 0.035-in. Glidewire (Terumo Interventional Systems). Both guidewires were replaced with two Advantage 0.014-in. guidewires (Terumo Interventional Systems), and two balloons for IVL were inserted (7 × 60 mm; Shockwave Medical Inc). Ballooning with IVL was simultaneously performed (complete treatment of 10 cycles for a total of 300 pulses; Fig 3) for the common iliac arteries. After IVL, relining of the old stents with balloon-expandable bare metal stents was performed (8 × 60 mm and 7 × 60 mm on the right side and 7 × 60 mm and 7 × 40 mm on the left side; Formula; Cook Medical Inc; Fig 4). Completion angiography revealed good patency of both iliac arteries without residual stenosis or dissection. Right femoral access was managed with a closure device (Perclose Prostyle; Abbott Cardiovascular), and the axillary access was then sutured in accordance with local practice.

**Fig 5:**
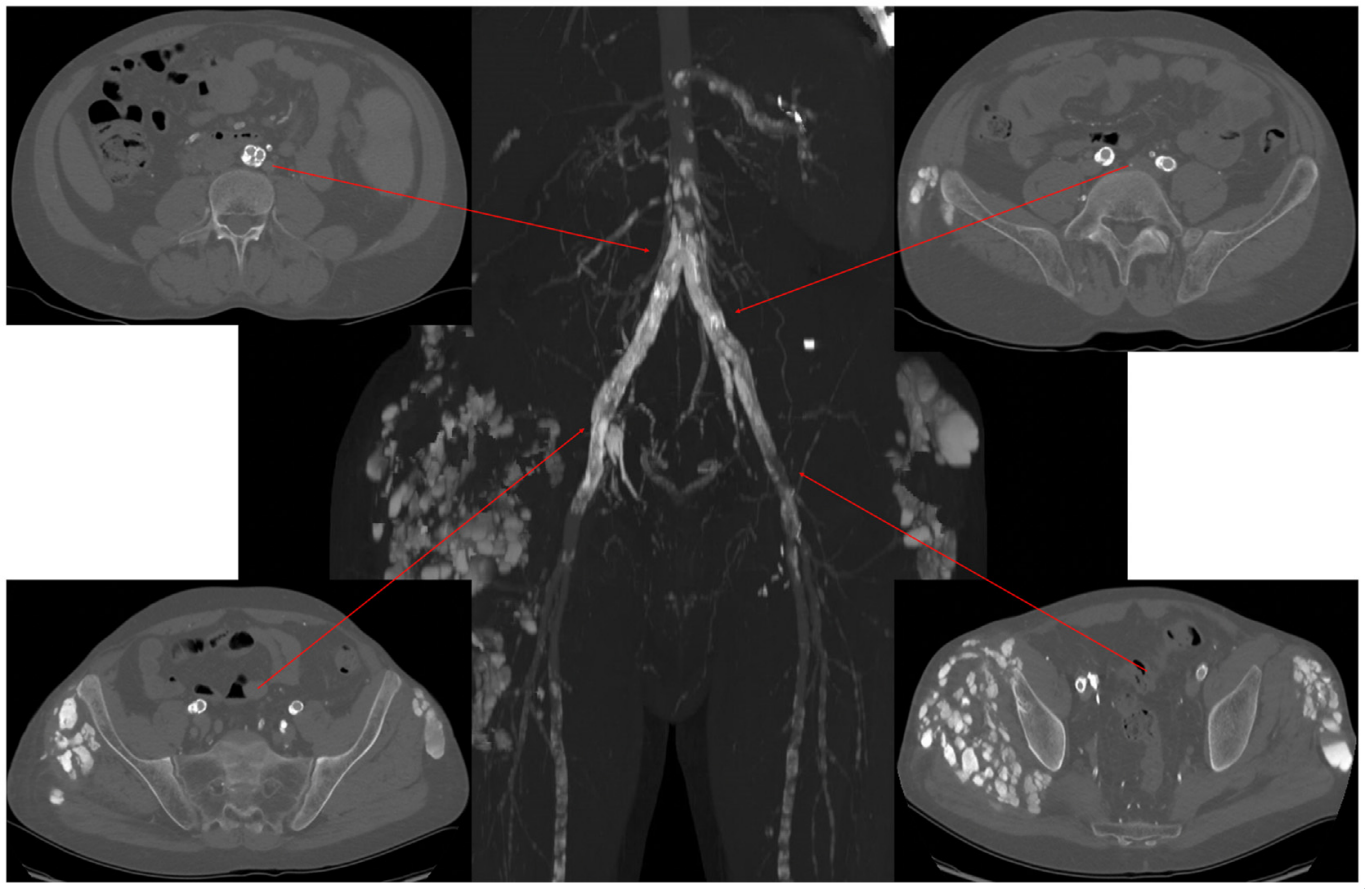
Three-month computed tomography (CT) scan.

The patient was discharged on the third postoperative day with dual antiplatelet therapy (aspirin and clopidogrel). His femoral pulses (3+ for both sides) and left radial pulse (4+) were palpable. At discharge, the toe brachial index was 0.7 on both feet, and no further procedures were performed. At the 3-month follow-up, the wounds on his feet had healed, and the patient did not have any pain. Follow-up CT angiography showed patent iliac arteries and satisfactory infrainguinal perfusion despite persistent diffuse wall calcifications (Fig 5). The stents placed in the iliac arteries had sufficient expansion and no signs of recurrent ISR (residual stenosis of 20% for both sides). Despite the absence of morphological changes in the iliac arteries compared with the preoperative CT findings, the compliance of the vessels had increased due to the mechanical disruption of the calcified plaque. At the 6-month follow-up, the femoral pulses (3+ for both sides) and left radial pulse (4+) were palpable. A duplex ultrasound scan detected multiphasic flow in both common femoral arteries. In addition, the patient did not experience intermittent claudication.

## Discussion

IVL is an effective and safe approach for the treatment of highly calcified obstructive disease in lower extremity arteries. In a recent meta-analysis, the investigators demonstrated that IVL is useful to reach a reduction in the stenosis grade of ≤60%.^4^

Regarding the iliac vessels, the Disrupt PAD III (Shockwave Medical peripheral lithoplasty system study for PAD [peripheral arterial disease]) study reported that IVL for calcified atherosclerotic lesions is a safe and effective option with the possibility of avoiding the use of a metal stent.^8^ In addition, IVL appears to be a safe and feasible option for endovascular treatment of severely calcified occlusions of the infrarenal aorta and aortic bifurcation.^9^ For the latter therapeutic option, two kissing IVL balloons should be used at the same time. Also, it is mandatory that both devices should be very to the atherosclerotic plaques to allow for strict, direct contact between the high-pressure ultrasonic energy and the calcium burden.

In the present case, we adopted the same technique with the use of two balloons. The choice to use an axillary access was related to the possibility to perform aortograms from the upper side and to inflate an aortic balloon in bailout situations. However, we were able to obtain intraluminal crossing of the iliac lesions without any risk of rupture during the whole procedure.

In addition, in our case, the iliac ISR was related to underexpansion of the bare metal stents previously placed into the common iliac arteries. This phenomenon is well known in the coronary arteries.^6^ However, an initial study about IVL of stent underexpansion in the superficial femoral artery has been recently reported.^7^ The action of IVL is only against calcium molecules. Thus, the presence of the stents should not affect the efficacy of the device. Future applications of IVL in the primary stenting of iliac lesions should be evaluated further to potentially reduce the risk of ISR during the follow-up period.

## Conclusions

The present case demonstrated the safety and effectiveness of IVL for the treatment of iliac ISR related to underexpansion of previously placed stents. The kissing ballooning technique should be mandatory to allow for strict contact of the devices close to the highly calcified plaques for calcium disruption. This technique should be used in very selected patients when other “standard” techniques cannot be used to obtain a satisfactory outcome. This technique requires further evaluation with multicenter experiences and adequate population sizes.

## Disclosures

None.
